# Moderating Effect of Cognitive Reserve on Brain Integrity and Cognitive Performance

**DOI:** 10.1093/geroni/igaa057.912

**Published:** 2020-12-16

**Authors:** Monica Nelson, Ross Andel, Julie Martinkova, Kateřina Čechová, Hana Marková, Jakub Hort

**Affiliations:** 1 University of South Florida, Tampa, Florida, United States; 2 Motol University Hospital, Prague, Czech Republic

## Abstract

Dementia is arguably the most devastating condition of older adulthood with treatment options still elusive. Alzheimer’s is the most prevalent form of dementia where cognitive deficits relate strongly to underlying brain pathology. However, there exist cases in which cognitive performance does not match the corresponding level of neuropathology. Attempts to explain this phenomenon often include the concept of cognitive reserve (CR), whereby greater CR (e.g., more education or higher occupational position) presumably results in less impairment relative to the extent of pathology early in disease progression but also greater impairment once cognitive symptoms manifest. We examined the influence of CR proxy variables (education and occupation) on the relationship between hippocampal volume and cognitive performance on tests of executive control and memory using data from the Czech Brain Aging Study (CBAS). Participants were cognitively normal/with subjective cognitive decline but without actual impairment (CN; n=115; M(age)=66.43; M(education)=15.90; 37 men) or had amnestic mild cognitive impairment (aMCI; n=165; M(age)=71.37; M(education)=14.92; 85 men). We found that hippocampal volume was significantly related to executive control (b=-.0001, p=.03) and memory (b=.0002, p<.001) for participants with aMCI, but only memory (b=.0002, p=.03) for CN participants. Occupational position moderated the association between memory and hippocampal volume in aMCI, with the result approaching significance (p=.07), whereby a greater link between memory problems and hippocampal atrophy was present in those previously in high occupational positions. No other moderations for occupational position or education emerged (ps>.25). We found evidence for the concept of CR using occupational position as proxy.

